# Early detection of stroke at the sudden sensorineural hearing loss stage

**DOI:** 10.3389/fneur.2023.1293102

**Published:** 2023-11-01

**Authors:** Yao Zhong, Hongyan Li, Gaifen Liu, Jia Liu, Jia-Jie Mo, Xingquan Zhao, Yi Ju

**Affiliations:** Beijing Tiantan Hospital, Capital Medical University, Beijing, China

**Keywords:** sudden sensorineural hearing loss, stroke, hearing loss, clinical feature, magnetic resonance imaging

## Abstract

**Background and purpose:**

Sudden sensorineural hearing loss (SSNHL) can be a prodromal symptom of ischemic stroke, especially posterior circulation strokes in the anterior inferior cerebellar artery (AICA) area. Early diagnosis and optimal treatment for vascular SSNHL provide an opportunity to prevent more extensive area infarction. The objective of our research was to find clues that suggest stroke at the stage of isolated sudden hearing loss.

**Methods:**

We retrospectively investigated the medical records of patients who received an initial diagnosis of sudden sensorineural hearing loss upon admission from January 2017 to December 2022 at Capital Medical University Affiliated Beijing Tiantan Hospital. Among these patients, 30 individuals who developed acute ischemic stroke during their hospital stay were enrolled as the case group. To create a control group, we matched individuals from the nonstroke idiopathic SSNHL patients to the case group in terms of age (±3 years old) at a ratio of 1:4. We collected the clinical characteristics, pure tone hearing threshold test results, and imaging information for all patients included in the study.

**Results:**

Three models were constructed to simulate different clinical situations and to identify vascular sudden sensorineural hearing loss (SSNHL). The results revealed that patients with SSNHL who had three or more stroke risk factors, bilateral hearing loss, moderately severe to total hearing loss, and any intracranial large artery stenosis and occlusion (≥50%) were at a higher risk of developing ischemic stroke during hospitalization. Consistent with previous studies, the presence of vertigo at onset also played a significant role in the early detection of upcoming stroke.

**Conclusion:**

Clinicians should be alert to SSNHL patients with bilateral hearing loss, moderately severe to total hearing loss and other aforementioned features. Early pure tone audiometric hearing assessment and vascular assessment are necessary for high-risk patients with SSNHL.

## 1. Introduction

Sudden sensorineural hearing loss (SSNHL), a subset of sensorineural hearing loss (SHL) (1), is widely regarded as one of the most common otolaryngological and potentially neurological emergencies (2). The incidence of SSNHL ranges from 5 per 100,000 to 150 per 100,000, primarily affecting individuals in the age group of 41–55 years, and the incidence has been rising in China in recent years (1, 3). Often, but not always, it is accompanied by vertigo and/or tinnitus. The pathogenesis and etiology of SSNHL remains unclear, and vascular mechanisms have gained more attention.

Previous studies indicated that 1.8%−4.2% of SSNHL patients were diagnosed with ischemic stroke, which was a higher percentage than that of people who were never diagnosed with SSNHL (4, 5). Several studies have demonstrated that sudden sensorineural hearing loss (SSNHL) is often a warning sign of an imminent stroke, particularly in the anterior inferior cerebellar artery (AICA) (4, 6–9). However, the diagnostic efficacy of HINTS (Head-Impulse-Nystagmus-Test-of-Skew) tests and HINTS-plus is compromised in these patients (10, 11). Clinical physicians currently do not possess efficient means to detect stroke during the SSNHL stage, specifically before the appearance of typical neurological symptoms and signs. This limitation can lead to a potential misdiagnosis.

Considering the time window for thrombolytic or interventional treatment, it is crucial to identify vascular SSNHL early before more devastating cerebral infarction. However, to our knowledge, there is still a lack of research on risk factors of stroke in isolated SSNHL patients. This case–control study aims to investigate the clinical characteristics, laboratory tests, auditory, and neuroimaging findings to facilitate the early identification of SSNHL related to stroke.

## 2. Methods

### 2.1. Study design and patients

Retrospectively, we investigated the medical records of patients who received an initial diagnosis of sudden sensorineural hearing loss upon admission from January 2017 to December 2022 at Capital Medical University Affiliated Beijing Tiantan Hospital. According to the Guidelines for Diagnosis and Treatment of Sudden Deafness 2015, China (3), the diagnostic standard of SSNHL in this study is rapidly evolving SHL with a minimum of 20 dB over at least two consecutive frequencies on tone audiometry that occurs within a period of 72 h. The inclusion criteria of the case group were as follows: age ≥18 years old; SSNHL as the first symptom (identified on pure tone audiometry on the day or second day of admission) and without neurological deficits at admission; development of focal neurological deficits (e.g., facial numbness and limb weakness, hemiataxia, dysarthria or Horner's syndrome) during hospitalization and diagnosis of acute ischemic stroke by magnetic resonance imaging (MRI) diffusion weighted imaging (DWI) or following computed tomography (CT, only for patients who are contraindicated for MRI); and complete medical records, laboratory test results and pure-tone audiometry records. The exclusion criteria included definite neurological deficits preceding SSNHL; no new focal neurological deficits during hospitalization but existing acute cerebral infarction on MRI-DWI; and hearing loss due to other specific diseases. Among all 1,882 patients with SSNHL on admission, 30 individuals (1.59%) met the above inclusion/exclusion criteria for the case group, which was checked and verified by two stroke specialists.

The controls were nonstroke idiopathic SSNHL inpatients in the same period (January 2017 to December 2022) as the case group. Patients with identifiable causes of hearing loss, such as noise-induced hearing loss, traumatic hearing loss, ototoxic drug poisoning, and Meniere's disease, were excluded. Given that atherosclerosis is commonly associated with advancing age (12), age-related hearing loss is the most prevalent type of auditory impairment (13). The control group was matched with each individual in the case group according to the patient's age (±3 years old) by random sampling. To optimize sample utilization and enhance the research efficiency to the maximum extent possible, each case had four age-matched controls. The control participants also underwent complete magnetic resonance imaging examinations and were examined by ENT specialists. Finally, a total of 150 patients were enrolled, with 30 patients in the case group and 120 patients in the control group. The standardized treatment plan for all of SSNHL patients in this study was based on the 2015 Guidelines for Sudden Deafness of China (10). Glucocorticoids and ginkgo leaf extract drop were the drugs mainly used in the treatment. Vasodilator prostaglandins, such as alprostadil, were not used.

### 2.2. Baseline characteristics and data collection

We edited a case report form (CRF) and digitized it using Epidata 3.1 software developed by EpiData Association in Copenhagen, Denmark. The study encompassed the collection of demographic and medical records from all 150 patients, including information on sex, age, hypertension, diabetes, hyperlipidaemia, history of stroke, and drinking and smoking history.

The detailed definitions of the six risk factors for stroke mentioned above are as follows: hypertension, indicated by a history of high blood pressure with a measurement of ≥140/90 mmHg as reported by the individual or diagnosed by a physician (14); diabetes, indicated by fasting plasma glucose levels of ≥7.0 mmol/L or taking glucose-lowering drugs (15); dyslipidaemia, deemed either self-reported physician-diagnosed hyperlipidaemia or the use of lipid-lowering drugs (16); stroke history, characterized by the sudden onset of focal neurologic deficits and diagnosed as either ischemic or haemorrhagic stroke; and smoking history, defined as smoking at least 1 cigarette per day for >6 months before admission (17); and drinking history, defined as consuming more than 100 ml of spirit alcohol more than three times a week based on self-report (18). Furthermore, accompanying symptoms and laboratory test results were also recorded. The detailed descriptions of the accompanying symptoms are as follows: dizziness, defined as the feeling of disturbed or impaired spatial orientation without a distorted or false sense of motion (19); vertigo, referring to the sensation of self-motion of the head or body, even when there is no actual movement, or the false perception that the visual surroundings are flowing or spinning (19); headache, defined as pain located above the orbitomeatal line (20); and tinnitus, defined as the perception of sounds that are not actually present (21).

### 2.3. Audiometric and neuroimaging assessments

The auditory assessment was ascertained by measuring pure tone averages (PTAs) from 500 Hz to 4,000 Hz for both bone and air conduction (ISO/EC17025), and the auditory assessments were performed by audiologists. According to the World Report on Hearing 2021, the degree of hearing loss was determined using the average hearing thresholds at 500 Hz, 1,000 Hz, 2,000 Hz, and 4,000 Hz. Hearing loss was classified as either mild to moderately severe (20–50 dB) or moderately severe to total hearing loss (≥50 dB) (22). Moreover, bilateral SSNHL was defined in this study as hearing loss in both ears that met the diagnostic criteria for SSNHL, and the grading of the audiometric assessment was based on the side with more severe hearing loss.

Brain MRI and magnetic resonance angiography (MRA) imaging were performed on all but one patient who had previously undergone coronary artery bypass graft surgery. However, one patient had typical focal neurological deficits (dysarthria and diplopia) after SSNHL, and subsequent CT (11 days later) showed hypodensity in the right brachium pontis. The interpretation of neuroimaging was independently achieved by a neurologist and a neuroradiologist to ensure accuracy and reliability. The vascular territories of infarct lesions in the case group were determined using MRI anatomical templates that have been previously validated for diagnosing arterial territories (23, 24). The distribution of lesions was classified into anterior circulation infarction (4/30), posterior circulation infarction (24/30) and border zone infarction (2/30). Furthermore, as per the New England Medical Center Posterior Circulation Registry, posterior circulation infarcts were classified into proximal (medulla and posterior inferior cerebellum), middle (pons and anterior inferior cerebellum), and distal territories (rostral brainstem, superior cerebellum, occipital and temporal lobes) (25).

In addition, we evaluated major intracranial large arteries in all 150 patients, including the internal carotid arteries (ICAs, intracranial segments), anterior cerebral arteries (ACAs, A1 segments), middle cerebral arteries (MCAs, M1–M3 segments), posterior cerebral arteries (PCAs, P1–P3 segments), vertebral arteries (VAs, intracranial segments), and basilar artery (BA). We used magnetic resonance angiography (MRA) with the WASID (Warfarin-Aspirin Symptomatic Intracranial Disease) criteria to measure the degree of intracranial artery stenosis (ICAS). This was graded as normal to mild stenosis (<50%) and moderate stenosis to occlusion (≥50%) (26).

### 2.4. Statistical analysis

SPSS version 26.0 (IBM, Armonk, NY, USA) was used for statistical analysis. We expressed dichotomous data as the numbers (percentages) and expressed continuous data as the median (Q1–Q3; interquartile range) or mean standard deviation. For continuous data, the Mann–Whitney U test or Student's *t* test was applied to compare data between the groups. The chi-square test was applied to analyse comparisons for dichotomous data; *p* < 0.05 was regarded as statistically significant. Three multivariate logistic regression models were constructed based on the different situations in clinical practice. Due to the limited sample size, we combined stroke risk factors such as “smoking history” from Table 1 into the variable of “Three or more stroke risk factors” to ensure the robustness of the fitted logistic model. Statistically significant variables in the clinical characteristics and laboratory tests were then included in the multifactor logistic regression analyses as model-1, simulating a scenario where patients did not receive a hearing test and head MRI scan in outpatient, emergency, or primary care settings. Model-2 was built upon model-1 and incorporated audiometry findings, simulating a situation where patients had undergone pure tone audiometry in the ENT clinic or ward but did not receive an MRI scan. Exploratory Model-3 added any large artery moderate stenosis to occlusion (≥50%) to Model-2. The 95% confidence intervals (95% CIs) and adjusted odds ratios (ORs) were calculated, and bilateral *p* values (*p* < 0.05) were assumed to be statistically significant. A receiver operating characteristic curve (ROC) was plotted, and the area under the ROC curve (AUC) was calculated.

**Table 1:** Demographics and clinical features.

**Variables**	**Control Group**	**Case Group**	**Total**	***P* value**
	**(*n* = 120)**	**(*n* = 30)**	**(*n* = 150)**	
**Demographic**				
Age (Mean ± SD)	56.67 ± 10.82	57.13 ± 10.64	56.76 ± 10.71	0.835
Male *n* (%)	62 (51.67)	25 (83.33)	87 (58.00)	**0.002**
**Medical history**				
Smoking History *n* (%)	25 (20.83)	19 (63.33)	44 (29.33)	<0.001
Drinking History *n* (%)	22 (18.33)	17 (56.67)	39 (26.00)	<0.001
Hypertension *n* (%)	39 (32.50)	20 (66.67)	59 (39.33)	<0.001
Diabetes mellitus *n* (%)	18 (15.00)	7 (23.33)	25 (16.67)	0.273
Dyslipidaemia *n* (%)	11 (9.17)	9 (30.00)	20 (13.33)	0.003
Stroke history *n* (%)	3 (2.50)	4 (13.33)	7 (4.67)	0.012
Three or more stroke risk factors *n* (%)	12 (10.00)	14 (46.67)	26 (17.33)	**<0.001**
Dizziness history *n* (%)	1 (0.83)	1 (3.33)	2 (1.33)	0.286
Tinnitus history *n* (%)	5 (4.17)	2 (6.67)	7 (4.67)	0.561
**Accompanying symptoms**				
Dizziness *n* (%)	55 (45.83)	17 (56.67)	72 (48.00)	0.288
Vertigo *n* (%)	25 (20.83)	13 (43.33)	38 (25.33)	**0.011**
Headache *n* (%)	3 (2.50)	3 (10.00)	6 (4.00)	0.061
Tinnitus *n* (%)	5 (4.17)	2 (6.67)	7 (4.67)	0.561

The variables marked in bold are clinically important and meaningful variables in univariate analysis.

## 3. Results

### 3.1. Baseline information

The case group consisted of twenty five males and five females, with an average age of 57.13 ± 10.64 years. The nonstroke control group consisted of 120 patients, comprised of 58 females and 62 males, with an average age of 56.67 ± 10.82 years. Table 1 presents a comparison of the baseline information. There were statistically significant differences between the two groups (*p* < 0.05) in terms of sex, dyslipidaemia, hypertension, stroke history, smoking, and drinking history. In the case group, 14 patients (46.67%) had three or more stroke risk factors, while in the control group, there were 12 patients (10%) with the same condition (*p* < 0.001). The incidence of vertigo in the case group was significantly higher (*p* = 0.011) than that in the control group among the accompanying symptoms. No significant differences were found between the two groups in terms of dizziness, headache, tinnitus, or other accompanying symptoms.

### 3.2. Audiometric findings and laboratory test findings

As depicted in Table 2, a comparison was conducted between the two groups regarding the hearing loss features. Concerning the side of hearing loss, there was a notable disparity in the proportion of bilateral SSNHL patients between the case group and the control group, with the former exhibiting a significantly higher proportion (*p* = 0.003). With respect to the degree of SSNHL, a significant distinction was observed in the range of moderately severe to total hearing loss (*p* = 0.017). Therefore, individuals with SSNHL displaying a degree of moderately severe to total HL have an increased likelihood of developing ischemic stroke. For lab tests, statistically significant differences (*p* < 0.001) were observed in serum creatinine (Scr). No significant difference was observed in the other indicators (*p* > 0.05).

**Table 2:** Audiometric, laboratory tests and neuroimaging findings.

**Variable**		**Control group**	**Case group**	**Total**	***p* value**
		**(*n* = 120)**	**(*n* = 30)**	**(*n* = 150)**	
**Pure tone audiometry** ***n*** **(%)**				
Side of hearing loss	Unilateral	115 (95.83)	24 (80.00)	139 (92.67)	**0.003**
	Bilateral	5 (4.17)	6 (20.00)	11 (7.33)	
Moderately severe to total hearing loss	No	43 (35.83)	4 (13.33)	47 (31.33)	**0.017**
	Yes	77 (64.17)	26 (86.67)	103 (68.67)	
**Laboratory data M (Q1, Q3)**				
HGB(g/L)		141.50 (131.0, 151.0)	147.00 (134.5, 155.3)	—-	0.147
LDL-C(mmol/L)		2.93 (2.5, 3.6)	2.95 (1.8, 3.8)	—-	0.618
AST (U/L)		17.15 (13.2, 21.6)	20.20 (14.6, 23.7)	—-	0.179
ALT (U/L)		20.05 (14.1, 30.0)	22.35 (16.8, 30.8)	—-	0.250
GFR (ml/min)		113.30 (103.9, 121.9)	107.73 (91.2, 121.5)	—-	0.310
Scr(μmol/L)		58.60 (49.5, 66.8)	70.45 (61.7, 82.5)	—-	**<0.001**
**Intracranial large arteries stenosis or occlusion(**≥**50%)*****n*** **(%)**				
Any intracranial large arteries		24 (20.00)	20 (66.67)	44 (29.33)	**<0.001**
Vertebrobasilar arteries		15 (45.50)	18 (60.00)	33 (22.00)	
Posterior cerebral arteries		8 (6.67)	4 (13.33)	12 (8.00)	
Middle cerebral arteries		2 (1.67)	4 (13.33)	6 (4.00)	

HGB, hemoglobin; LDL-C, low-density lipoprotein cholesterol; ALT, alanine transaminase; AST, aspartate aminotransferase; GFR, glomerular filtration rate; Scr, serum creatinine; vertebrobasilar artery, vertebral arteries and/or basilar artery.

### 3.3. Neuroimaging findings

Among the individuals in the case group, the infarcts involved the cerebellum in seventeen cases, pons in nine cases, corona radiata in five cases, occipital lobe in four cases, frontotemporal lobe in three cases, corpus callosum in three cases, medulla oblongata in two cases, midbrain in one case, and thalamus in two cases. Table 3 shows the vascular territory of the infarct lesions in the case group. There were 24 cases (80%) of posterior circulation infarction (PCI), with significantly higher rates than anterior circulation (4, 13.3%) and border zone infarction (2, 6.6%). Specifically, the middle territory of PCI was most often involved, either as an isolated infarct or in combination with other territory infarcts. Regarding to hearing loss features in different arterial territories of case group, there was no significant difference between the PCI and anterior circulation or border zone, as illustrated in Supplementary material.

**Table 3:** Distribution of infarcts in the case group.

**Arterial territories of ischemic lesions**	***n* = 30**
**Posterior circulation territories**	24 (80.0%)
Distal territory alone	2 (6.6%)
Middle territory alone	8 (26.6)
Proximal territory alone	3 (10%)
Middle territory + proximal and/or distal territory	11 (36.7%)
**Border zone and anterior circulation territories**	6 (20.0%)
Border zone	2 (6.6%)
Middle cerebral arteries supply area	4 (13.3%)

For the assessment of intracranial arteries, 22 of 30 (73.3%) patients in the case group had intracranial large artery stenosis or occlusion, among whom 20 (66.6%) patients had ≥50% stenosis or occlusion, including 18 (60.0%) cases presenting with ≥50% stenosis or occlusion in the vertebral arteries and/or basilar artery. Additionally, out of the 120 patients in the nonstroke control group, 24 individuals (20%) were found to have moderate to severe stenosis or occlusion in their large arteries, with a statistically significant difference (p <0.001).

### 3.4. Multivariate logistic regression analysis

To improve clinical applicability, three distinct multivariate logistic regression models were designed to determine the risk factors associated with ischemic stroke in patients with sudden sensorineural hearing loss (SSNHL).

#### Model 1: clinical presentation

For this primary model, the hypothesized scenarios were that patients did not undergo pure tone audiometry and MRI during their clinic visits. Patients with three or more stroke risk factors showed a markedly higher risk of ischemic stroke (adjusted OR 4.974; 95% CI 1.659–14.918; *p* = 0.004). Vertigo, when present at onset, was a significant risk factor of stroke-related SSNHL (adjusted OR 2.846; 95% CI 1.031–7.857; *p* = 0.044). Similarly, patients with higher serum creatinine levels on admission were at a greater risk of stroke (adjusted OR 4.974; 95% CI 1.659–14.918; *p* = 0.004), as shown in Table 4.

**Table 4:** Multivariate regression logistic models in different practical situations.

**Variables**	**Crude OR (95% CIs)**	**Model-1**	**Model-2**	**Model-3**
		**Adjusted OR (95%CIs)**	**Adjusted OR (95%CIs)**	**Adjusted OR (95%CIs)**
Male	0.214 (0.077, 0.596)^**^	0.753 (0.227, 2.499)	0.816 (0.232, 2.869)	0.631 (0.157, 2.542)
With three or more stroke risk factors	7.875 (3.098, 20.016)^***^	4.974 (1.659, 14.918)^**^	4.967 (1.563, 15.784)^**^	3.913 (1.101, 13.905)^*^
Accompanying Vertigo	2.906 (1.247, 6.771)^**^	2.846 (1.031, 7.857)^*^	3.754 (1.250, 11.275)^*^	3.393 (1.022, 11.262)^*^
SCr(μmol/L)	1.065 (1.031, 1.100)^***^	1.053 (1.016, 1.092)^**^	1.061 (1.021, 1.103)^**^	1.057 (1.020, 1.096)^**^
Binaural hearing loss	5.750 (1.622, 20.387)^**^	—	8.040 (1.694, 38.153)^**^	6.823 (1.301, 35.783)^*^
Moderately severe to total HL	3.630 (1.188, 11.090)^*^	—	5.219 (1.214, 22.431)^*^	5.613 (1.192, 26.425)^*^
Any large arteries stenosis or occlusion(≥50%)	8.000(3.315, 19.308)^***^	—	**–**	7.264 (2.403, 21.961)^***^

OR, odds ratio; CI, confidence interval; Scr, serum creatinine; HL, hearing loss.

Model-1: Clinical presentation and laboratory data (simulating a scenario without audiometry and neuroimaging).

Model-2: Model-1+ pure tone audiometric (simulating a scenario without neuroimaging).

Model-3: Model-2+ any intracranial large artery stenosis or occlusion (≥50%).

^*^p <0.05; ^**^p <0.01; ^***^p <0.001.

#### Model 2: audiometric findings

For cases where complete audiometry was carried out, findings related to the side and degree of hearing loss were integrated into Model-1. Audiometric data indicated that both bilateral hearing loss (adjusted OR 8.040; 95% CI 1.694–38.153; *p* = 0.009) and moderate to severe hearing loss (adjusted OR 5.219; 95% CI 1.214–22.431; *p* = 0.026) were associated with an increased risk of stroke.

#### Model 3: MRI findings

Building on Model-2, the presence of intracranial artery stenosis or occlusion (≥50%) was introduced to develop Model-3. Factoring in intracranial artery issues became a strong risk factor for evolving into cerebral infarction during hospitalization (adjusted OR 7.264; 95% CI 2.403–21.961; *p* < 0.001).

**Figure 1:**
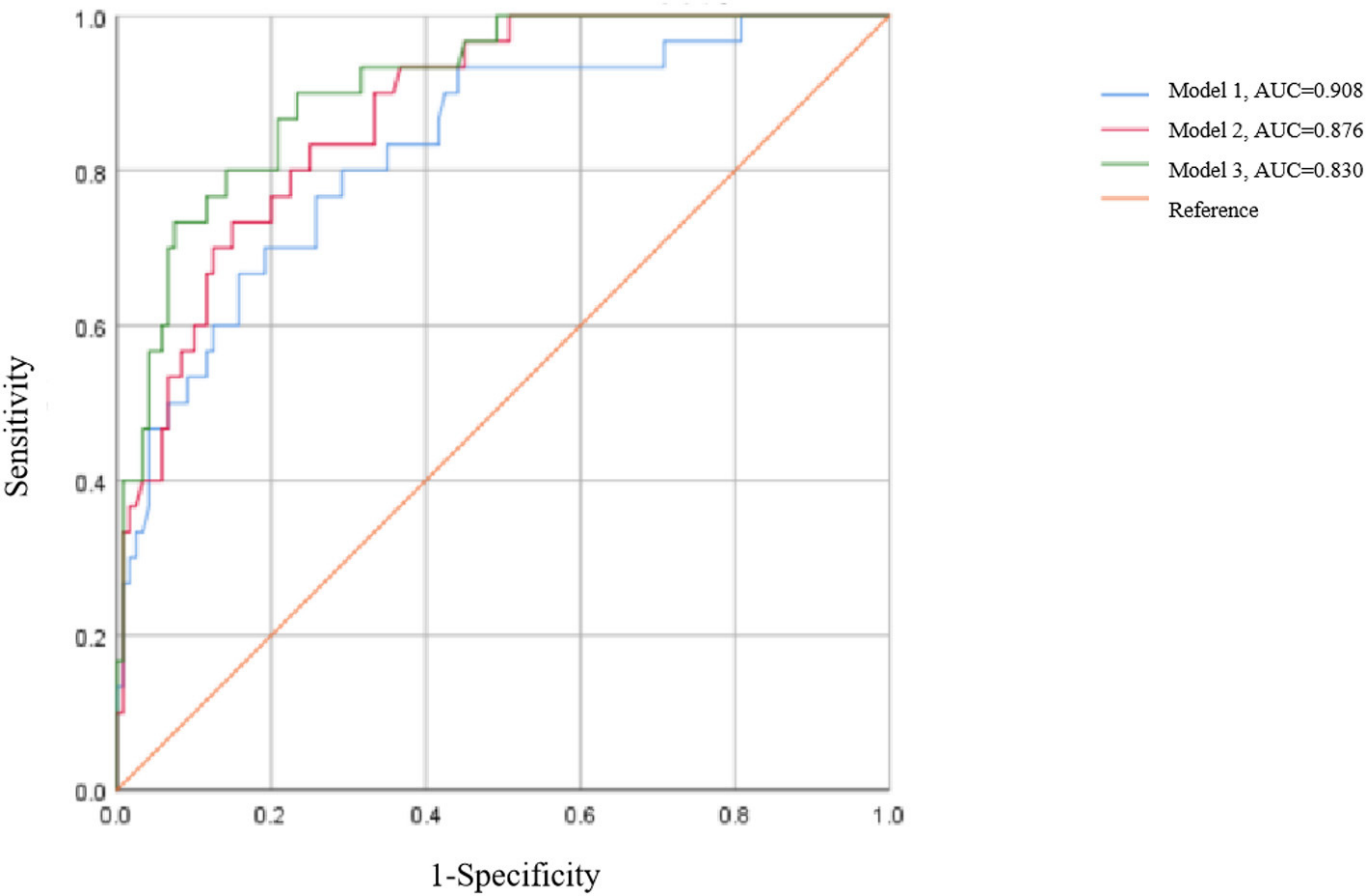
Receiver operating characteristic curves (ROC) of the three models.

The effectiveness of each model was gauged through receiver operating characteristic curves (Figure 1). Of all the models, Model-3 showed the highest predictive precision with an area under the curve (AUC) value of 0.908, followed by Model-2 (0.876) and Model-1 (0.830).

### 3.5. Illustrative cases

**Figure 2:**
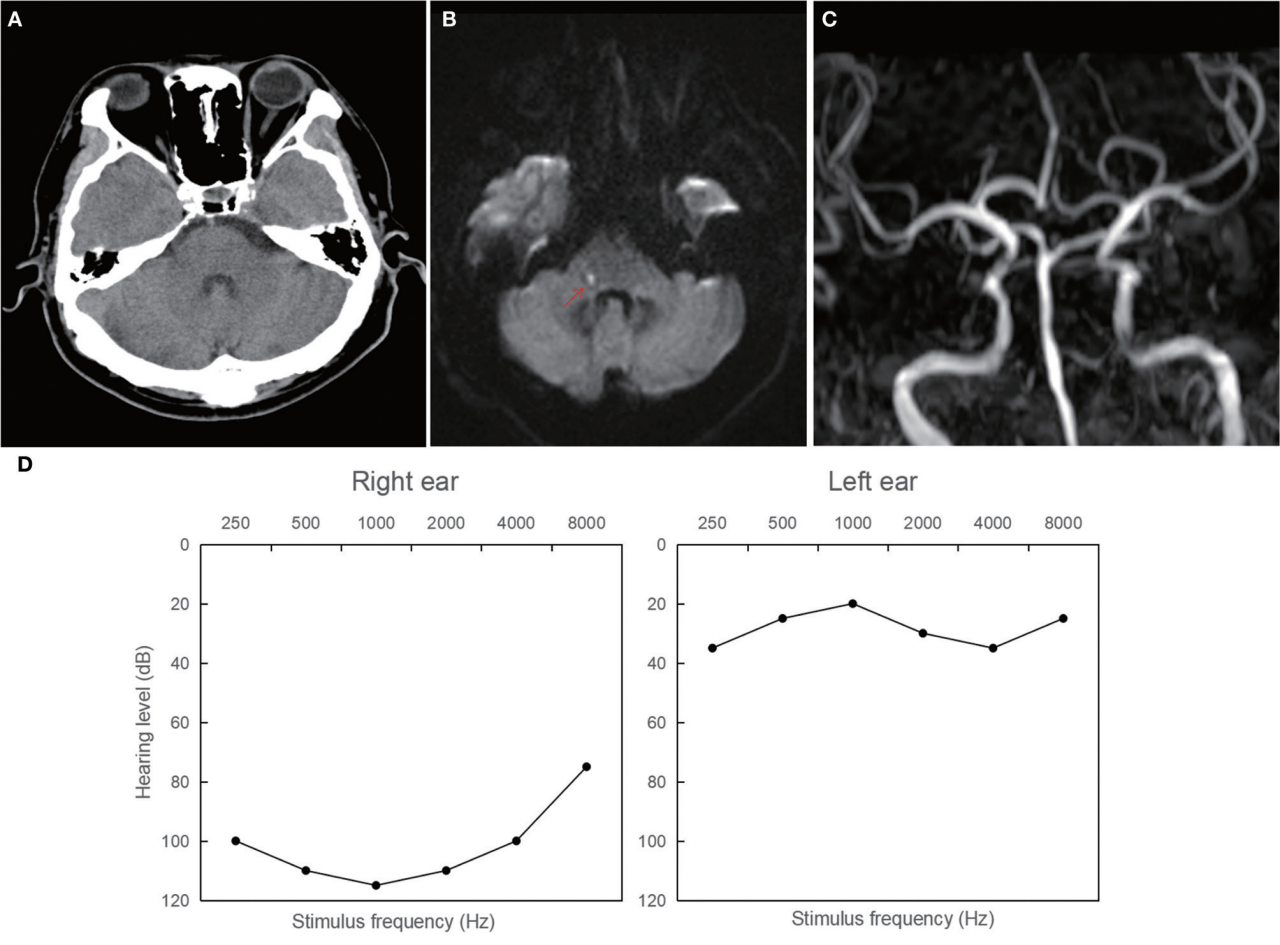
Neuroimaging and audiological findings in patient 1. **(A)** The initial normal CT. **(B)** Magnetic resonance imaging of the brain showing an acute ischemic lesion localized on the right dorsolateral pontine region. **(C)** Magnetic resonance angiography showings stenosis of the right intracranial vertebral artery and a moderate stenotic lesion in the mid-segment of the basilar artery. **(D)** Pure tone audiometry documenting profound sensorineural hearing impairment on the right ear (108.75 dB).

Patient 1: a 42-year-old male, who had a medical history significant for essential hypertension and type II diabetes mellitus, presented with acute unilateral right-sided sensorineural hearing loss, concomitant vertigo, and tinnitus. The initial cranial CT performed in the emergency department demonstrated no acute intracranial pathology, as visualized in Figure 2A. On the fifth day of hospitalization, he developed clinical signs of right-sided central facial nerve palsy and hypoesthesia to thermal and nociceptive stimuli over the facial region. A subsequent MRI of the brain revealed an acute ischemic infarct localized on the right dorsolateral pontine region, as evidenced in Figure 2B. Magnetic resonance angiography (MRA) corroborated significant vascular pathology, with pronounced stenosis of the right intracranial vertebral artery and a moderate stenotic lesion in the mid-segment of the basilar artery, as detailed in Figure 2C. Audiometric evaluation using pure tone audiometry documented profound sensorineural hearing impairment on the right, registering at 108.75 dB.

**Figure 3:**
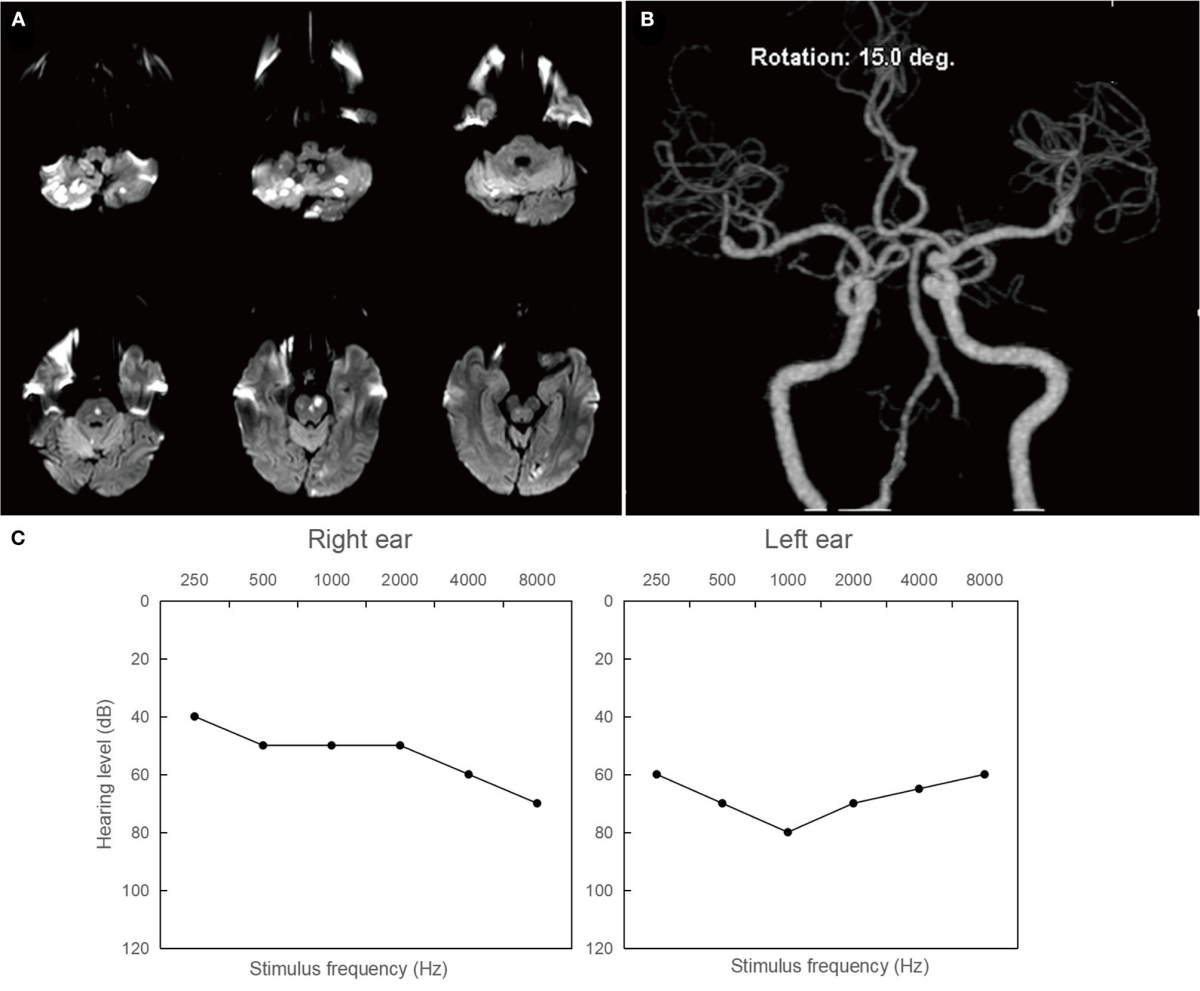
Neuroimaging and audiological findings in patient 2. **(A)** Diffusion weighted imaging of the brain showing hyperintense lesions involving in the bilateral cerebellum, pons, and left occipital lobe. **(B)** Magnetic resonance angiography showings multiple stenoses in the bilateral vertebral arteries and basal artery. **(C)** Pure tone air conduction audiograms illustrating severe hearing loss (71.25 dB) in the left ear and moderately severe hearing loss (52.5 dB) in the right ear.

Patient 2: A 63-year-old male presented with intermittent dizziness for seven days and bilateral hearing loss for 3 days prior to admission. Two days after being admitted, the patient developed weakness in his left lower extremity (grade 4/5). MRI of the brain revealed acute infarction in the bilateral cerebellum, pons, and left occipital lobe (Figure 3A). Magnetic resonance angiography showed multiple stenoses in the bilateral vertebral arteries and basal artery (Figure 3B), indicating that the condition was likely caused by an artery-to-artery embolism. Figure 3C illustrates severe hearing loss (71.25 dB) in the patient's left ear and moderately severe hearing loss (52.5 dB) in the right ear.

## 4. Discussion

### 4.1. Association of accompanying vertigo with stroke

Regardless of the inclusion or exclusion of audiometric and neuroimaging variables in model-1 to model-3, the presence of accompanying vertigo consistently serves as a dependable risk factor for stroke. Similarly, Tzu-Pu Chang et al. found that individuals who presented with both sudden hearing loss and vertigo had a higher likelihood of stroke than those with either vertigo or sudden hearing loss alone. Furthermore, there seemed to be an increasing risk of stroke as the interval between episodes of these two symptoms diminished (27). This may be relevant to the inner ear anatomical structure and its associated blood supply. Fundamentally, internal auditory arteries (IAAs), which supply the inner ear, originating from the AICA (45.4%), superior cerebellar artery (24.4%), posterior inferior cerebellar arteries (5.4%) and even basilar artery (16%), as well as other anastomotic branches (6.7%), are extremely sensitive to ischaemia and hypoxia (28–30). The common cochlear artery and anterior vestibular artery are branches of the IAA, and simple ischaemia of the common cochlear artery or anterior vestibular artery may cause isolated hearing loss or vertigo. Compared with the common cochlear artery, the IAA is closer to the larger branch of the vertebrobasilar artery. Therefore, ischaemia of the IAA is more likely to indicate local stenosis or occlusion of the large vessels rather than impairment of the inner ear microcirculation. Furthermore, an animal study (31) demonstrated that the medial vestibular nucleus is more susceptible to ischaemia than other structures in the brainstem and cerebellum. Therefore, accompanying vertigo may also be attributed to dorsolateral medulla damage caused by vertebrobasilar system ischaemia.

### 4.2. Hearing loss features and stroke

Bilateral SSNHL is rare, accounting for approximately 5% of all SSNHL cases, and is often associated with poor prognosis (32). Our study demonstrates that there is a significant correlation between bilateral SSNHL and potential ischemic stroke. Hence, it highlights the importance of standardized hearing assessments in clinical practice, especially in a scenario when MRI has not yet been applied (Model-2). Case reports and case series suggest that bilateral SSNHL is associated with posterior circulation infarction (33–35). However, due to the lack of a control group and the relatively small sample size, those results have been considered less convincing. A previous study analyzed the characteristics of patients who had bilateral SHL with occlusion of the vertebrobasilar artery and showed that the patients often suffered early vertigo and delayed neurological deficits (36). The findings are similar to those of our study, but the relation between bilateral hearing loss and stroke-related SSNHL has not been explored in the past. The mechanism may be bilateral inner ear ischaemia caused by local stenosis or occlusion of the inferior 1/3 of the BA where bilateral AICA or IAA emanates or, rarely, bilateral vertebral artery stenosis (37), resulting in damage to inner ear hair cells that are hypersensitive to hypoxia and ischaemia. Further advancements in vascular examination techniques are required to accurately detect stenoses or occlusions from the AICA or PICA to the IAA to ascertain the hypothesis.

Apart from which side hearing loss occurs, the multivariate logistic model-2 and model-3 showed that the severity of hearingloss was a risk indicator for stroke. A previous study showed that individuals with more severe hearing loss tend to have a higher CHADS2 score, suggesting that the severity of hearing loss was positively related to the degree of atherosclerosis (38). Another study showed that individuals with moderate to severe hearing loss were more likely to have a history of stroke; but there is no statistically significant correlation between moderate to severe hearing loss and occurrence of stroke 5 years later (39). There may be a potential link between the degree of hearing loss and the occurrence of stroke. However, the ability to predict long-term stroke occurrence based on this association is currently insufficient. To our knowledge, our study is the first to explore the link between the degree and side of hearing loss and the incidence of short-term stroke in SSNHL patients. For patients presenting with moderate to severe bilateral hearing loss, it is necessary to conduct brain magnetic resonance imaging (MRI) and assess the cranial large vessels (Model-3). Further research with a larger sample size is required to further study the correlation between the type of hearing loss and the likelihood of stroke.

### 4.3. Intracranial large artery stenosis and ischemic stroke in SSNHL patients

In contrast to previous studies that exclusively examined patients with anterior inferior cerebellar artery infarction (40–42), our study concentrates on patients with SSNHL, especially those who developed ischemic stroke in the hospital. Among the case group, the middle territory (19/30, 63.33%) of posterior circulation vascular territories (24/30, 80%) was the most common position of infarcts. There were significantly more people with moderate to severe intracranial artery stenosis in the case group (20/30, 66.67%) than in the control group (24/120, 20%). First, this result demonstrates the significance of vertebrobasilar artery ischaemia, specifically the basilar artery and its branches in the middle territory, as a major concern for patients with suspected vascular SSNHL. It is crucial for clinicians to pay more attention to large artery conditions, as shown in model-3, particularly in arteries such as the vertebral arteries or basilar artery, even in the absence of typical neurological symptoms and signs. In addition, anterior circulation (4/30, 13.33%) and border zone (2/30, 6.66%) stroke can also be upcoming events in SSNHL patients, whose pathogenesis remains unknown. Previous studies have demonstrated that hypoperfusion of the anterior inferior cerebellar artery (AICA) can potentially cause episodic sudden sensorineural hearing loss (SSNHL), not only as a prodrome of AICA or PICA territory infarcts but also in other infarct cases (43). In this study, 50% of patients (3 out of 6) with anterior circulation or border zone infarction also had posterior circulation vascular stenosis or occlusion. The first symptom of hearing loss may be attributed to inadequate blood supply in the labyrinthine as a warning signal of global cerebral hypoperfusion at the same time. However, there are currently no effective methods to confirm inner ear ischaemia or infarction. Future studies with larger sample sizes and with more comprehensive examinations of the inner ear are needed. Moreover, among the few patients (2/30, 6.67%) who did not exhibit major intracranial artery stenosis, it is worth considering and investigating the possibility of cardiogenic or extracranial vascular embolism.

This study represents the initial endeavor to explore the risk of stroke among patients with sudden sensorineural hearing loss (SSNHL) during their hospitalization. In cases where there is no noticeable neurological deficit or it appears too late, thrombolytic or antithrombotic treatment may not be feasible. This study provides clues to this apparent conundrum. We conducted an exploration to build three models simulating different clinical situations, incorporating clinical characteristics, laboratory tests, pure tone audiometry, and neuroimaging. These models aim to assist clinicians and serve as a reference for future research. The innovative findings reveal that bilateral sudden sensorineural hearing loss and moderately severe to total hearing loss are independent risk factors for subsequent stroke. Additionally, our study supports earlier findings indicating that individuals with SSNHL accompanied by vertigo at onset are at an elevated risk of stroke (27, 44).

### 4.4. Limitations

Despite our study's implications, we acknowledge its limitations. First, this study is a retrospective case–control study with a limited sample size and thus is at risk of information bias. Second, pure tone audiometry was measured as the average of 500, 1,000, 2,000, and 4,000 Hz. Quartering is a common method of hearing calculation and may not be sufficient to assess hearing loss at low or high frequencies alone; therefore, additional studies in patients with high-tone or low-tone hearing loss are needed. Third, due to the retrospective nature of this study, detailed time information between SSNHL and neurological deficits was not fully collected. As a result, the study was unable to determine the ideal time point for magnetic resonance imaging. There is still a need for further sample size expansion and multicentre validation in the future.

## 5. Conclusion

We cautiously consider that SSNHL may not be the direct cause of stroke but rather a potential indicator or warning sign, particularly for posterior circulation stroke in the middle territory, during the progression of the disease. According to our research, clinicians should be alert to patients with three or more stroke risk factors, bilateral SSNHL, and moderately severe to total hearing loss, effectively screen high-risk vascular SSNHL groups and complete brain structure and vascular imaging evaluations.

## Data availability statement

The raw data supporting the conclusions of this article will be made available by the authors, without undue reservation.

## Ethics statement

Ethical approval was not required for the study involving humans in accordance with the local legislation and institutional requirements. Written informed consent to participate in this study was not required from the participants or the participants' legal guardians/next of kin in accordance with the national legislation and the institutional requirements.

## Author contributions

YZ: Investigation, Writing—original draft, Data curation, Methodology. HL: Writing—review & editing, Investigation. GL: Data curation, Writing—review & editing. JL: Formal analysis, Writing—review & editing. J-JM: Methodology, Writing—review & editing. XZ: Conceptualization, Funding acquisition, Writing—review & editing. YJ: Conceptualization, Funding acquisition, Methodology, Supervision, Writing—review & editing.
