# Supplementary material for: Early detection of stroke at the sudden sensorineural hearing loss stage
Source: Front Neurol. 2023 Nov 1;14:1293102. doi: 10.3389/fneur.2023.1293102 (PMC10646485; doi:10.3389/fneur.2023.1293102)
Supplement: Supplementary file 1 [file Table_1.pdf]

Table 1. Time from hearing loss to MRI finding of acute infarction

Patient/no/age/sex	Time interval (day/days)
1/52/M	4
2/34/M	2
3/52/M	21
4/63/F	6
5/65/F	4
6/35/F	3
7/67/M	6
8/48/M	4
9/61/M	6
10/64/F	8
11/43/M	5
12/59/M	2
13/61/M	8
14/59/M	6
15/69/M	2
16/49/M	3
17/51/M	4
18/46/M	16
19/50/M	7
20/46/M	4
21/71/M	6
22/50/M	16
23/57/M	8
24/63/M	2
25/73/M	*
26/71/M	6
27/58/M	11
28/76/M	5
29/62/F	6
30/59/M	1

*This patient were contraindicated for MRI and follow-up brain CT demonstrated infarction in brachium pontis.

Table 2. Hearing loss features in different arterial territories of case group

Variables		Arterial territories of ischemic lesions		p value
		Posterior circulation territories(n=24)	Border zone and anterior circulation territories(n=6)	
Pure tone average		74.69±26.68	86.67±18.57	0.311
Side of hearing loss	Unilateral	19(79.17)	5(83.33)	0.819
	Bilateral	5(20.83)	1(16.67)	
Moderately severe to total hearing loss	No	4(16.67)	0(0.00)	0.283
	Yes	20(83.33)	6(100.00)	

Table 3. Multivariate regression logistic models

Variables	OR value	95% CIs	p value
Male	1.076	0.199 ~ 5.824	0.932
Smoking history	2.724	0.603 ~ 12.298	0.193
Drinking history	2.578	0.521 ~ 12.758	0.246
Hypertension	1.755	0.482 ~ 6.387	0.393
Dyslipidaemia	6.869	1.049 ~ 44.962	**0.044**
Stroke history	6.875	0.519 ~ 91.118	0.144
Accompanying Vertigo	5.448	1.405 ~ 21.126	**0.014**
Binaural hearing loss	9.787	1.365 ~ 70.165	**0.023**
Moderately severe to total HL	8.554	1.361 ~ 53.750	**0.022**
Scr(μmol/L)	1.061	1.017 ~ 1.106	**0.006**
Any large arteries stenosis or occlusion（≥50%）	7.021	2.006 ~ 24.574	**0.002**

Notes: OR, odds ratio; CI, confidence interval; HL, hearing loss; Scr, serum creatinine
